# *Ficus sycomorus* extract reversed behavioral impairment and brain oxidative stress induced by unpredictable chronic mild stress in rats

**DOI:** 10.1186/s12906-017-2012-9

**Published:** 2017-11-28

**Authors:** Harquin Simplice Harquin Foyet, Serge Tchinda Deffo, Pascaline Koagne Yewo, Iulia Antioch, Stéphane Zingue, Emmanuel Acha Asongalem, Pierre Kamtchouing, Alin Ciobica

**Affiliations:** 1 Department of Biological Sciences, Faculty of Science, University of Maroua, Cameroon, P.O. Box: 814, Maroua, Cameroon; 2 Department of Life and Earth Sciences, Higher Teachers’ Training College, University of Maroua, Cameroon, P.O. Box: 55, Maroua, Cameroon; 3 Department of Research, Faculty of Biology, Alexandru Ioan Cuza University, 11 Carol I Blvd, 700506 Iasi, Romania; 4 Department of Biomedical Sciences, Faculty of Health Sciences, University of Buea, Cameroon, P.O. Box 63, Buea, Cameroon; 5 Department of Animal Biology and physiology, Faculty of Science, University of Yaounde I, Yaounde, Cameroon

**Keywords:** *Ficus sycomorus*, Mood disorders, Memory, Unpredictable chronic mild stress

## Abstract

**Background:**

Stress, regardless of its nature is nowadays recognized as one of the major risk factors for neuropsychiatric diseases, such as mood and anxiety disorders. The brain compared with other organs is more vulnerable to oxidative damage mainly due to its high rate of oxygen consumption, abundant lipid content, and relative insufficiency of antioxidant enzymes. Thus, the identification of neural mechanisms underlying resistance and vulnerability to stress is of crucial importance in understanding the pathophysiology of neuropsychiatric disorders and in developing new treatments, since the existing ones are for several reasons subject to increasing limitations. This study was aimed to assess the effects of hydromethanolic extract of *Ficus sycomorus* stem bark on depression, anxiety and memory impairment induced by unpredictable chronic mild stress (UCMS) in rats.

**Methods:**

These effects were studied using anxiety-related behavior, depression-related behavior, anhedonia-like behavior and the Y maze task. Sucrose test was performed twice (before and after UCMS) to assess anhedonia in rats. Liquid chromatography-mass spectrometry analysis of the extract were performed. The antioxidant activities of the extract were assessed using total glutathione (GSH) content and malondialdehyde (MDA) level (lipid peroxidation) in the rat temporal lobe homogenates.

**Results:**

The extract of *F. sycomorus* in a dose of 100 mg/kg significantly increased the sucrose consumption and the swimming time which had been reduced by the unpredictable chronic mild stress (*p* < 0.001). The extract also significantly reduced (*p* < 0.01) the latency time in the novelty-suppressed feeding test. In the elevated plus-maze, the extract (100 and 200 mg/kg) significantly reduced (p < 0.01) the time and the number of entries into the closed arms. The treatment with the extracts also significantly increased alternation in the Y-maze (p < 0.01 for 100 mg/kg). The extract significantly increased the total GSH content and reduced MDA level in rat temporal lobe. For the LC–MS analysis, the major compound in the extract was a flavonoid with formula C_22_H_28_O_14_.

**Conclusions:**

*F. sycomorus* reversed the harmful effects of UCMS on mood and behaviors in rats and it possesses an antidepressant property that is at least in part mediated through the oxidative pathway.

## Background

Through its mediators, stress can lead to acute or chronic pathological, physical and mental conditions in individuals with a vulnerable genetic, constitutional and/or epigenetic background [1]. Acute stress may trigger panic attacks and psychotic episodes wile chronic stress may cause physical, behavioral and/or neuropsychiatric manifestations including anxiety, depression, bipolar disorders, executive and/or cognitive dysfunction. Neuropsychiatric manifestations are the cause of high mortality rate across the world. About 450 million persons suffer from mental disturbances or from disturbances of behavior [2] and about 6.8 million people die every year as a result of neurological disorders [3]. About 4% of the populations in developed countries are affected by general anxiety disorder [4]. Precisely, in Africa, about 7.9 to 10% of the population suffer from depression and anxiety disorders, respectively [5]. According to a report of the Cameroonian Ministry of Health, in Cameroon, a prevalence of 7% of mortality rate is due to psychiatric disorders [6].

Neuropsychiatric troubles are multifactorial disorders with a multitude of causes, such as environmental causes, stress, genetic, molecular and biochemical alterations. Recent epidemiological data show that chronic stress condition is highly associated to psychiatric mental health impairment: burnout, mood disorders (anxiety, depression), sleep disorders, behavioral disorders (drug addiction, alcohol) [7].

Modelling of human neuropsychiatric disorders in animals is extremely challenging given the subjective nature of many symptoms, the lack of biomarkers and objective diagnostic tests, and the early state of the relevant neurobiology and genetics. However, our group demonstrated in the last 10 years that oxidative stress is implicated in neurological and psychiatric disorders including Parkinson disease, Alzheimer disease, depression, anxiety disorders, schizophrenia and bipolar disorder [8–10].

Many drugs are useful in the treatment of neuropsychiatric diseases but secondary effects, inaccessibility for some patients, and the high cost of these products for the poor population makes their use problematic [5]. The therapy of neuropsychiatric diseases is traditionally done with the use of different parts of curative plants. Nevertheless, these treatments are made using decoctions and infusions of these plants or mixtures of several plants [11, 12].

One of the most commonly used plant in the therapy of mental disorders in sub-Saharan Africa is the sycamore fig or *Ficus sycomorus*. *F. sycomorus* belongs to the family Moraceae. It is a tree of the northern savannahs, which can reach up to 30 m [12]. Its curative proprieties are due to abundant active secondary metabolites such as flavonoids [13] and polyphenols [3]. Previous studies on *F. sycomorus* extracts shows antibacterial effects [14]; a relaxant activity on muscles and anaesthetic properties [15]; antioxidant and antidiabetic properties [16–19]; anticonvulsants and antiepileptic properties [16]. The population traditionally use *F. sycomorus* for the treatment of behavioral manifestations related to anxiety and depression. However, no scientific study was made to confirm or to infirm this use of *F. sycomorus*. In a recent study, Tijani et al. [20] showed that the methanolic extract of the bark of this plant has a significant anxiolytic activity when tested in three behavioral models: Elevated plus maze, Elevated zero maze and Open field test.

Using the stem bark of *Morus alba*, a plant of the same family, Muhammad et al. [21, 22] indicated an high correlation and regression between phenolic contents and antioxidant potentials of the extracts, which effective free radical inhibition and CNS-depressant activity. It is now widely known that there is a high rate of comorbid anxiety and depressive disorders in the population. More than 70% of individuals with depressive disorders also have anxiety symptoms [23]. In the other hand, similar to other Moraceae, the stem bark extract of this plant possesses anticonvulsant activity. It appeared reasonable for us to explore the effect of this extract with both anxiolytic and anticonvulsant activities in some animal models of depression. We thus used the unpredictable chronic mild stress model which is considered by many laboratories to be the animal model of depression that has the greatest validity and translational potential [24, 25].

In this context, in the present study we decided to determine the effect of the hydromethanolic extract of the stem bark of *F. sycomorus* on depression, anxiety and amnesia caused by chronic mild unpredictable stress on rats. The bioactive compound were assess by the LC-MS, the antidepressant effect and anxiolytic effect were studied in sucrose consumption test and in elevated plus maze test while the effect on memory was investigated in Y-maze test. Since sucrose preference is attenuated by a diversity of chronic stressors, including chronic mild and unpredictable stress, it was performed twice (before and after UCMS) to assess anhedonic effects in rats. The antioxidant activities of the extract using total glutathione (GSH) content and Lipid peroxidation (measured as malondialdehyde; MDA) in the brain homogenate were taken as reliable indicators for the contribution of free radical generation in oxidative stress.

## Methods

### Plant material and extraction

*Ficus sycomorus* stem bark was collected at Zockok Latdeo (Far North Region, Cameroon) Cameroon (longitude E 10 ° 57′ 389"; latitude N 014° 24' 172"; altitude 428 m).in July 2015, and identified by Dr. Souaré Konsala, a plant taxonomist of the Faculty of Sciences, University of Maroua. A Voucher specimen was deposited under the number 27006/HNC for further verification. For the extraction, the barks of *F. sycomorus* were dried shadow during 16 days then they were crushed in a mill. 2 kg of powder was mixed with 6 L of a hydromethanolic mixture made of 70% water and 30% methanol (*V*/V) during 72 h with daily stirring. The mixture was filtered using Wathman filter paper no. 3 and the filtrate concentrated in the rotavapor for the extraction of the methanol. The concentrated solution was freezed and lyophilized for the extraction of water and 140 g of raw extract were obtained.

### Experimental animals and groups

A total of 30 male Wistar rats weighing 200 to 240 g were purchased from the Laboratory of animal physiology of the University of Yaounde I. The animals were housed in polypropylene cages (38 × 23 × 10 cm) with not more than six animals per cage, at 25 ± 2 °C and humidity of 45% ± 5% under a 12-h light/dark cycle (lights on at 6:00 a.m., lights off at 6:00 p.m.) and received standard diet and water ad libitum. The animals were housed for 2 weeks under controlled conditions for acclimatization before the experiments. The animals were randomly assigned in to 5 groups of 6 animals each: control group with no stress (treated with saline), CUMS group (exposed to chronic stress and treated with saline only), CUMS + imipramine 20 mg/kg, CUMS + *F. sycomorus* extract 100 mg/kg, CUMS + *F. sycomorus* extract 200 mg/kg. The doses were fixed based on earlier unpublished studies on the hydroalcoholic extract of *F. sycomorus* extract in our laboratory. All efforts were done to minimize the number of animals used in the experiment. The experimental procedures were conducted in accordance with NIH-Care and Use of Laboratory Animals manual (8th Edition) and were approved by the Local Bioethics Committee (Faculty of Science, University of Maroua, Cameroun; document Ref. N°14/0261/Uma/D/FS/VD-RC).

### Preliminary phytochemical screening and high-resolution mass spectra

The preliminary phytochemical screening of the *F. sycomorus* stem bark was carried out according to the method described by Trease and Evans [19].

The high resolution mass spectrometric screening of the extracts of *F. sycomorus* stem bark was carried out using an Orbitrap Fusion tribrid mass spectrometer. The system was equipped with an Agilent HPLC system consisting of LC-pump, PDA detector, auto-sampler and a column compartment. The ESI source was operated in positive ion mode. Nitrogen was employed as sheath gas (30 arbitrary units) and auxiliary gas (10 arbitrary units). The heated capillary was set to 275 °C. The separations were performed using a Macherey & Nagel Nucleodur C18 Gravity column (3 μm, 2 × 125 mm2) at 40 °C. Solvent A was a mixture of 0.1% formic acid in water and solvent B was 0.1% formic acid in acetonitrile. The flow rate was set to 400 mL/min. Samples were analyzed using a gradient program as given: 5% solvent B for 1 min; followed by a linear gradient to 60% B within 24 min and a fast flushing step to 95% B within 2 min was conducted. After 5 min at 95% solvent B the start conditions were repositioned and the column was equilibrated for 6 min for the consecutive injection. 1H and 13C NMR spectra respectively all correlation experiments were recorded with a Bruker Avance 600 spectrometer equipped with a TCI CryoProbe. Optical rotations were recorded by means of a PerkinElmer polarimeter 341 in MeOH at 20 °C.

### Unpredictable chronic mild stress procedure

The induction of chronic stress on the rats was done according to the method used by Frih et al. [26]. All groups of UCMS were submitted to 30 days of chronic mild stress. During this period, several stressors have been used: food deprivation, water deprivation, forced swimming, flashing light, isolation, wet litter and wet litter at 4 °C. The chronology and the length of some stressors change every day, in order to minimize its predictability. Normal control rats were kept intact in their cages for 30 days, receiving only usual care, food and water. The program stressors and daily duration are listed in Table 1.

**Table 1:** Program of stressors and duration applied every day. During all these days the saline solution, plant extract or imipramine were administrated depending on the group

Day	Stressors	Duration
1	Water deprivation	24 h
2	Food deprivation	24 h
3–4	Isolation	48 h
5	Flashing light	3 h
6	Food deprivation	24 h
7	Forced swimming 20 °C	10 min
8	Wet litter	1 h
9	Water deprivation	24 h
10	no stress	–
11	Wet litter 4 °C	2 h
12	Flashing light	1 h 30 min
13	Food deprivation	24 h
14	Forced swimming 20 °C	15 min
15–16	Isolation	48 h
17	Water deprivation	24 h
18	Food deprivation	24 h
19	Flashing light	2 h
20	Wet litter	1 h
21–22	Isolation	48 h
23	no stress	–
24	Forced swimming 20 °C	10 min
25	Wet litter 4 °C	2 h
26	Food deprivation	24 h
27	Flashing light	2 h
28	Isolation	24 h
29	Water deprivation	24 h
30	Wet litter	1 h

#### Behavioral studies

**Fig. 1:**
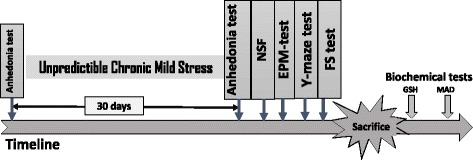
Experimental design. Baseline sucrose-preference was assessed before beginning the chronic unpredictable mild stress (CUMS). Only the animals having reduced the preference for sucrose by more than 50% were selected for the next steps of the experiment. *F. sycomorus* extract or imipramine was administered daily by gavage during both the 30 days stress sessions and behavior tests. At the end of the stress period, behavioral tests were carried out in a growing of stress order. The same set of animals was used for all the behavioral tests: day 31 for NSF, EPM, Y-maze and days 32, 33 for FST. Following the FS test, the mice were sacrificed for biochemical assay

On the last day of the CUMS protocol, the animals’ behavioral activities were tracked and recorded using trial version of ANY-maze 4.9 behavioral software. The order of the behavioral tests is shown in Fig. 1.

##### Sucrose preference consumption test

The sucrose-preference test was carried out before and at the end of the 30 days UCMS exposure. Anhedonia, measured as reduced preference for sweet solutions constitutes, with social avoidance or isolation, a feature closely related to the state of defeat in animals [27]. By developing this test before and after the UCMS, it is possible at the end of the stress period to establish that the animals are in an anhedonic state characterized by a significant decrease in their sucrose intake. The test was performed as described by Wilmer et al. [28]. Briefly, rats were first trained to experience and drink sucrose solution (1%) for 24 h, by presenting them simultaneously with two identical bottles, one with sucrose solution (1%) and the other tap water. In the testing protocol, rats were given a free choice between the two bottles for 24 h. The position of the bottles was switched at the mid-point of testing to prevent possible effects of side preference in drinking behavior. No deprivation of food was applied either before or during the test. The consumption of the solution of sucrose and of water was estimated at the same time in control groups and experimental groups by weighing bottles. The difference in their respective initial and final weights was calculated. The preference for sucrose was calculated according to the following formula:

$$ \% sucrose preference=\frac{\mathbf{Sucroseintake}\;\left(\mathbf{g}\right)}{\mathbf{Sucroseintake}\ \left(\mathbf{g}\right)+\mathbf{Water}\mathbf{intake}\ \left(\mathbf{g}\right)}X\;\mathbf{100} $$

##### Novelty-suppressed feeding test

The novelty-suppressed feeding test use in our experiment was similar to the version used by Kristen et al. [27]. After 24 h of food deprivation, a single food pellet (regular chow) was placed in the center of the open field arena (72 × 72 × 36 cm^3^). Each rat was then placed in a corner of the arena and the latency to bite was recorded using a video camera. Immediately afterwards, the rat was returned to its home cage and the amount of food consumed during the subsequent 5 min was measured (home food consumption).

##### Elevated plus maze (EPM)

The elevated plus maze test is used to measure the degree of anxiety in rodents [29–31]. Our device was constituted of four arms (50 × 10 cm), two open arms perpendicular to two closed arms with 16 cm high walls. The intersection of the four arms (central area) is a square of 10 × 10 cm. The apparatus was elevated of 50 cm from the ground. 30 min after oral administration of the DMSO solution, imipramine or the extract, every animal was put in the centre of the platform facing an open arm and allowed to explore maze freely during 5 min. Since the rats fears the empty and high spaces, his exploration of open arms shows a less anxious behavior. On the contrary, the more the animal remains in the closed arms, his behavior are known to be anxious. The 5 min sequences were recorded by a video camera to measure the following parameters: Open arms duration, closed arms duration, open and closed arms entries, head dipping frequency, rearing frequency and grooming duration. After each assay, the maze was carefully cleaned with 70° ethanol to remove residual odor.

##### Y- maze test

Short-term memory was assessed by spontaneous alternation behavior in the Y-maze task. The Y-maze used in the present study consisted of three arms (35 cm long, 25 cm high and 10 cm wide), carrying mention A, B and C respectively, with an equilateral triangular central area. 30 min after the last administration of the tested compounds, each rat was placed at the end of one arm and allowed to move freely through the maze for 8 min. The movements of animals in the maze was video tracked. The first 3 min were considered as period of habituation in the maze and the behavioral parameters was considered only during the 5 remaining minutes. An arm entry was counted when the hind paws of the rat were completely within the arm. Spontaneous alternation behavior was defined as entry into all three arms on consecutive choices. The number of maximum spontaneous alternation behaviors was then the total number of arms entered minus 2 and percent spontaneous alternation was calculated using the following formula [32].

$$ \mathbf{Pourcentage}\mathbf{of}\mathbf{alternation}=\frac{total number of alternations}{maximal number of altenations}\times \mathbf{100} $$

Spontaneous alternation behavior is considered to reflect spatial working memory, which is a form of short-term memory.

##### Forced swimming test (FST)

The forced swimming test was conducted based on the method of Arbabi et al. [33]. The apparatus consisted of a plastic cylinder (25 cm diameter × 50 cm height) filled with 30 cm deep water at 24 °C ± 2 °C. At the end of UCMS protocol, pretest was conducted for 15 min. The animals were individually allowed to swim for 15 min in the swim tank. After the pretest session, the animals were dried with a towel and placed under a heat lamp for 10 min to avoid hypothermia and returned to their respective cages. The water was changed after a trial with each animal to avoid influence to next animal. After 24 h, same procedure was followed to conduct the test swim session for 5 min. The top view of the activity was recorded with a video camera mounted on the ceiling of the behavior test room. The recorded videos were scored by an observer blind to the treatment regimen and duration of immobility was calculated using a stop watch.

### Biochemical parameter assay

After the behavioral tests, all rats were deeply anesthetized (using sodium pentobarbital, 100 mg/kg b.w., i.p., Sigma-Aldrich, Germany), decapitated and whole brains were removed and, weighed and homogenized (1:10) with Potter Homogenizer in ice-cold 0.1 M potassium phosphate buffer (pH 7.4), 1.15% KCl. The homogenate was centrifuged (15 min at 960×g) and the supernatant was used for assays of total content of MDA and reduced GSH levels as previously described by Wilbur et al. [34] and Ellman [35], respectively.

### Statistical analysis

All the results were expressed as mean ± SEM. The data were analyzed by one-way ANOVA followed by Newman-Keuls’s multiple comparison test as the post hoc test. All analyses were performed using the software Graph Pad Prism version 6.00 for Windows, Graph Pad Software, San Diego, California, USA, http://www.graphpad.com/. Results were considered significant at *p* < 0.05.

## Results

### Phytochemical screening

The phytochemical screening of the hydromethanolic extract revealed the presence of flavonoids, saponins, tannins, triterpenes, cardiac glycosides and phenols.

### LC-MS results

**Fig. 2:**
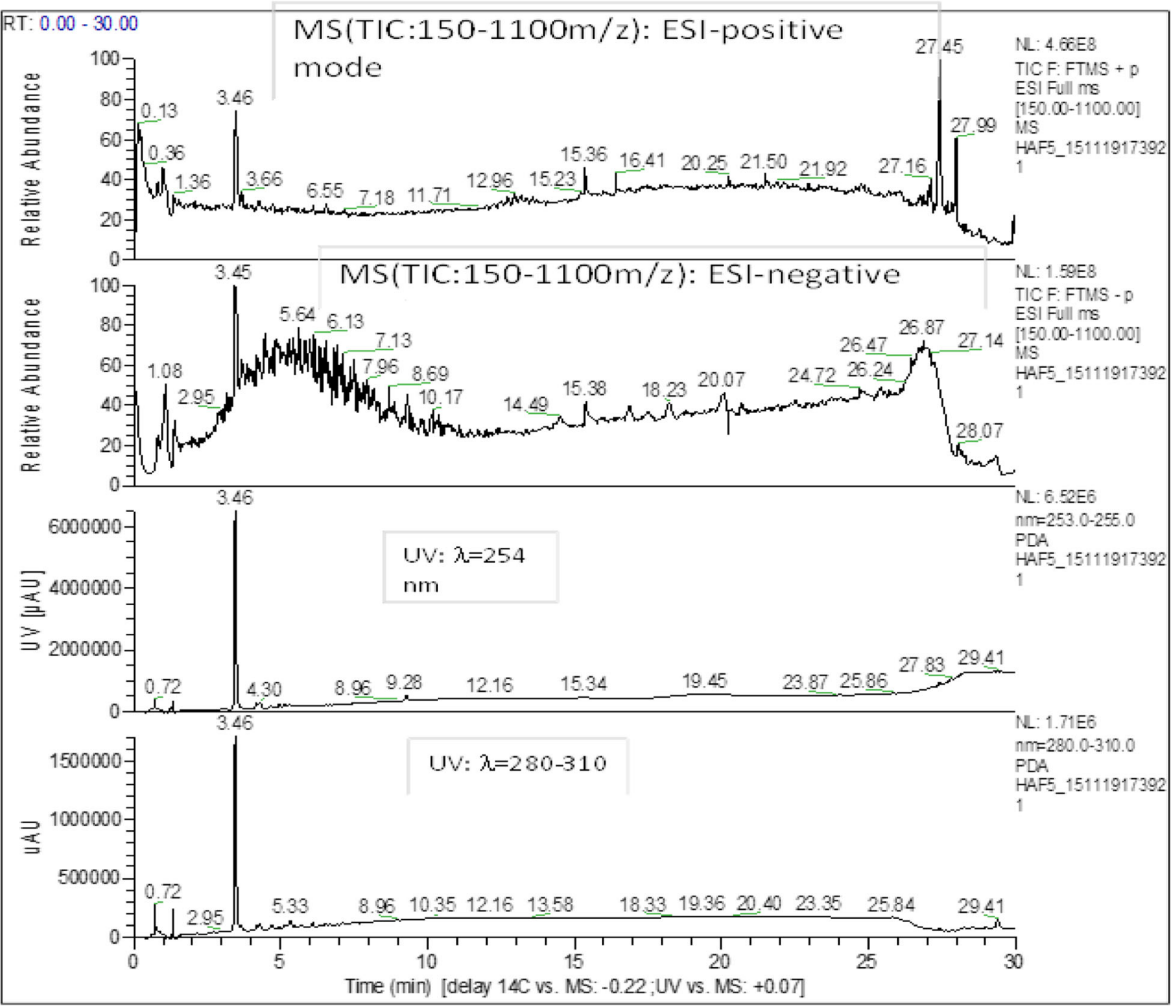
Full scan chromatogram of the hydromethanolic extract of *F. sycomorus*

**Fig. 3:**
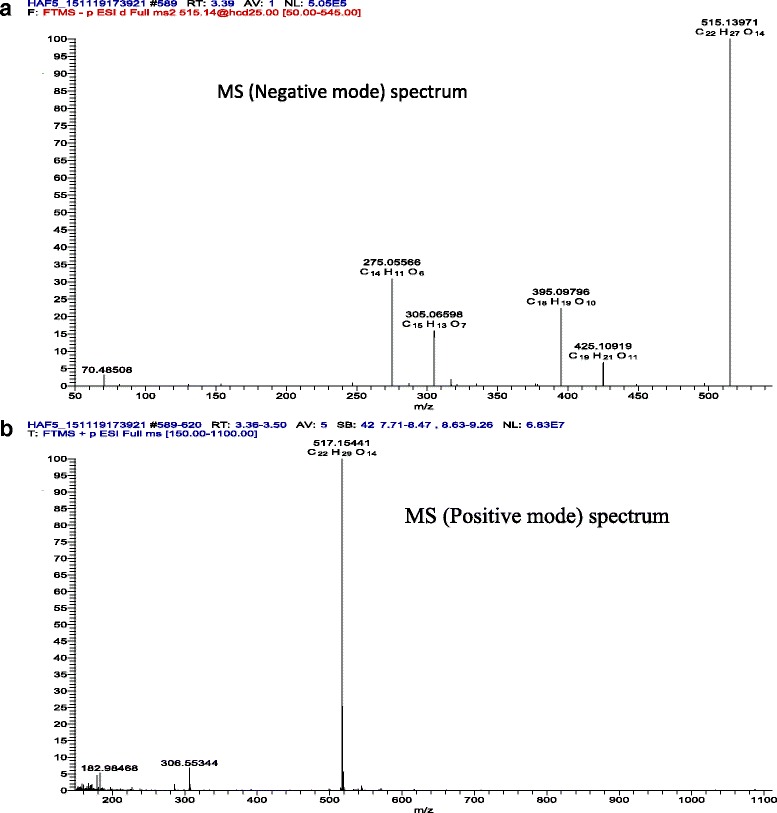
Mass spectrometry of the hydromethanolic extract of *F. sycomorus.* **a**: negative mode (Unitary mass 515, 14 g; brute formula: C_22_H_27_O_14_); **b**: positive mode *(*Unitary mass 517, 15 g; brute formula: C_22_H_29_O_14_)

The LC-MS analysis in positive mode (ES+), has shown the presence of two substances having close retention time. It is a major compound and a minor one. The major compound with a peak of retention (RT) at 3.45-3.46 min, very polar, is a glycoside flavonoid, with two sugars seeing, it’s mass (515 g), it’s abundant absorption at λ = 254, 280-310 nm and it’s twelve oxygen atoms. The comparison with data base has indicated that is racemosic acid with formula: C_22_H_28_O_14_ (Fig. 2 and Fig. 3).

### Effect of hydromethanolic extract of *F. sycomorus* on body weight in rats

**Fig. 4:**
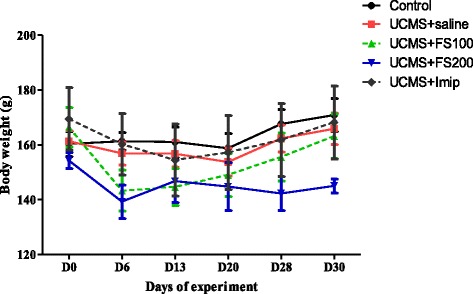
Effect of hydromethanolic extract of *F. sycomorus* on body weight (g) in rat

The variation in the weight of the experimental animals during the experiment is shown in Fig. 4. During the first week of unpredictable chronic mild stress, there was a general decrease in the body weight of all animals except those in the control group. After two weeks, the weight of the animals of the control, UCMS group and those treated with the extracts increased. ANOVA coupled with the Newman-Keuls t-test showed no significant increase in the body weight of the animals relative to their weight on day 0 or in relation to the control group.

### Effect of hydromethanolic extract of *F. sycomorus* on anhedonia in rats

#### Effect of *Ficus sycomorus* on sucrose 1% consumption

**Fig. 5:**
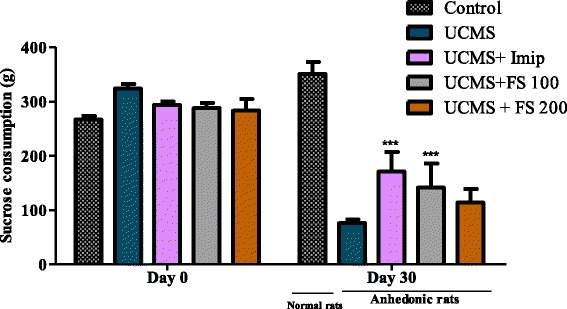
Effect of hydromethanolic extract of *F. sycomorus* on anhedonia. Sucrose consumption before (Day 0) and after (Day 30). UCMS: unpredictable chronic mild stress; Imip: imipramine, FS: *Ficus sycomorus* extract. *** *P* < 0,001 vs. equivalent animal group at day 0

The Fig. 5 represents graphs of sucrose consumption before and after the unpredictable chronic mild stress. The quantity of sucrose consumed was about 300 g of sucrose per group before UCMS in all groups. The UCMS has considerably reduced the quantity of sucrose consumed in all the animals when compared to the control group. During the UCMS, the sucrose consumption of animals treated with *F. sycomorus* extract (100 mg/kg) as well as imipramine (20 mg/kg) significantly increased. (*P* < 0,001).

#### Effect of *Ficus sycomorus* on the percentage of sucrose preference

**Fig. 6:**
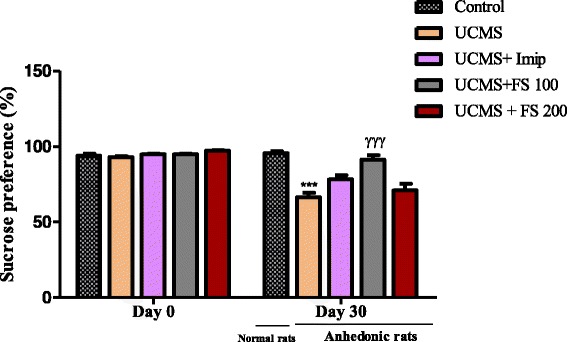
Percentage of sucrose preference in rats before the UCMS (Day 0) and the effect of hydromethanolic extract of *F. sycomorus* on anhedonia (Day 30). UCMS: unpredictable chronic mild stress; Imip: imipramine, FS: *Ficus sycomorus* extract. *** P < 0,001 vs. Control; ^γγγ^ *p* < 0,001 vs. anhedonic rats treated with saline only

Figure 6 represents the percentage of sucrose preference in rats before the UCMS (A) and the effect of hydromethanolic extract of *F. sycomorus* on anhedonia (B). Before the UCMS sucrose preference was relatively very high (about 95%) in every animal group. After the UCMS, we noted a significant decrease of this percentage in all animals of the negative control group compared to normal animals. The treatment with Ficus extracts at a dose of 100 mg/kg has significantly increased (*p* < 0.001) the preference in sucrose for animals of Ficus 100 group. Nevertheless, those treated with Ficus at a dose of 200 mg/kg and treated with imipramine presented a non-significant increase of the preference in sucrose compared to stressed but non-treated animals.

### Effect of hydromethanolic extract of *Ficus sycomorus* on the latency in the forced swimming test

**Fig. 7:**
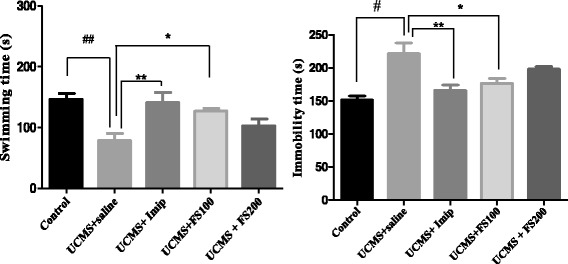
Effect of the hydromethanolic extract of *F. sycomorus* on swimming and immobility time of rat before and after unpredictable chronic mild stress. UCMS: unpredictable chronic mild stress; Imip: imipramine, FS: *F. sycomorus* extract. #*p* < 0.05; ##*p* < 0.01 vs. control; *p < 0.05; ** p < 0.01; *** *p* < 0.001 vs. anhedonic rats treated with saline only

One way ANOVA showed a significant effect on immobility [F (4, 25) = 8.84; *p* = 0.00013] and on swimming [F (4, 19) = 5.77; *p* = 0.0032] among different treatment groups. Newman-Keuls Multiple Comparison Test indicated that the extract at the dose 100 mg/kg and imipramine (20 mg/kg) significantly caused a reduction in immobility and an increase in swimming when compared to UCMS- animal treated with saline only (*p* < 0.01 and *p* < 0.05respectively) (Fig. 7).

### Effect of hydromethanolic extract of *Ficus sycomorus* on the latency in the novelty supressed feeding test.

**Fig. 8:**
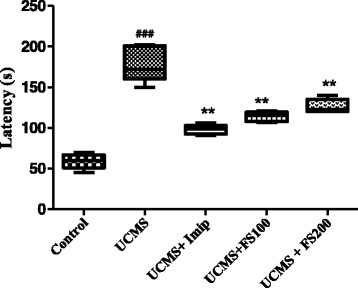
Effect of hydromethanolic extract of *F. sycomorus* on the latency in the novelty-suppressed feeding test in control and CUMS rats. Values are mean ± SEM (*n* = 6). ###*P* < 0.001 compared to control. **P < 0.01 compared to CUMS group

The CUMS group exhibited a longer latency to feed in the novelty-suppressed feeding test than the control group (Fig. 8). One-way ANOVA showed a significant effect of the extract on latency [F (4, 20) = 66.67, *p* < 0.0357]. Newman-Keuls Multiple Comparison Test indicated a significant reduction in latency with all the doses of the extract used as compare to UCMS group (p < 0.01).

**Fig. 9:**
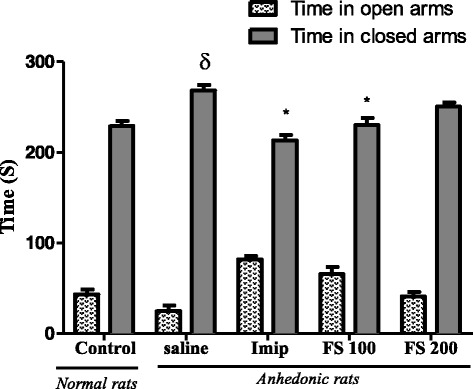
Effect of *F. sycomorus* extract on the time spend in open and closed arms. Values are mean ± SEM (n = 6). *P < 0.05 compared to control. ^γ^P < 0, 05 vs. anhedonic rats treated with saline only

**Fig. 10:**
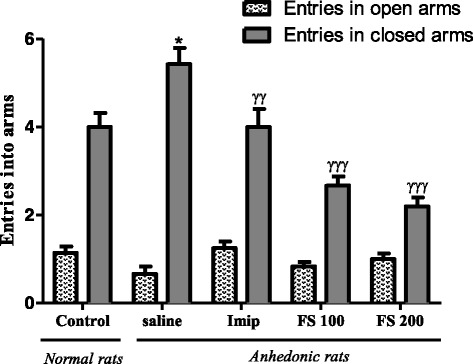
Effect of the hydromethanolic extract of *F. sycomorus* on the number of entries in open and closed arms. Imip: imipramine, FS: *Ficus sycomorus* extract. * *P* < 0, 05; ^γγ^ *p* < 0, 01, ^γγγ^ *p* < 0,001 vs. Anhedonic rats treated with saline

**Fig. 11:**
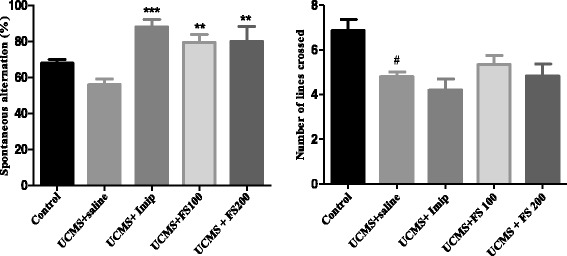
Effect of the hydromethanolic extract of *F. sycomorus* on the percentage of spontaneous alternation and on locomotor activity of rats in the Y-maze. TNn = normal witness; TNg = negative witness; Imip = imipramine. ** P < 0.01; * p < 0.05 vs. TNn; ^γ^p < 0.05 vs. TNg

**Fig. 12:**
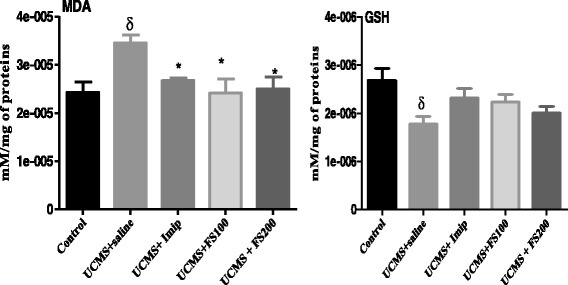
Effect of *F. sycomorus* extract on total content of reduced GSH and MDA levels of the brain homogenate before and after unpredictable chronic mild stress.). UCMS: unpredictable chronic mild stress; Imip: imipramine, FS: *Ficus sycomorus* extract. *** P < 0,001 vs. equivalent animal group at day 0

**Fig. 13:**
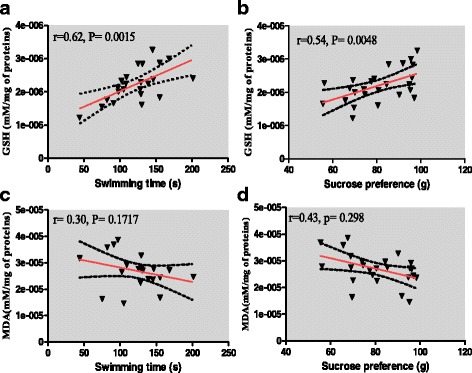
Pearson correlation between swimming time vs. GSH (**a**), Sucrose preference vs. GSH (**b**), swimming time vs. MAD (**c**) and Sucrose preference vs. MAD (**d**); with the 95% confidence band of the best-fit line

### Effect of the hydromethanolic extracts of *Ficus sycomorus* on anxiety

#### Effect of the hydromethanolic extract of *Ficus sycomorus* on the time spent in the opened and closed arms of the EPM

The time spent in the opened arm by animals of the negative control group significantly decreased (*p* < 0.05) when compared to that of normal animals. This time is significantly (*p* < 0.01) higher for animals treated with *Ficus sycomorus* extract at a dose of 100 mg/kg compared to the negative and positive control groups. For animals treated with a higher dose of plant extract (200 mg/kg), the time made in the opened arm is increased compare to that of the negative witness group but deceased in comparison to that of the group treated with imipramine 20 mg/kg, these differences were however non-significant. The time done in the closed arm by the negative witness group significantly increased (*p* < 0.001) when compare to that of the normal witness group but significantly decreased (*p* < 0.05) when compare to that of animals treated with *Ficus* extracts (100 mg/kg) as well as those treated with imipramine (20 mg/kg) (Fig. 9).

#### Effect of the hydromethanolic extract of *Ficus sycomorus* on the number of entrance in the opened and closed arms of the EPM

The UCMS has reduced the number of entry in the opened arms and increased the number of entrance in the closed arms in a significant way (p < 0.05). The treatment with *F. sycomorus* extracts in big dose (200 mg/kg) has significantly increased the number of entrances in the opened arms meanwhile the treatment with imipramine has increased more significantly (*p* < 0.01). The treatment with *F. sycomorus* extracts in small dose has increased in a non-significant way the number of entries in the opened arms. On the other hand, the use of the two extracts for the treatment of chronically depressed animals has significantly (*p* < 0.001) reduced the number of entries in the closed arm of the elevated plus maze. The treatment with imipramine has significantly reduced (*p* < 0.01) this number but not as much as treatment with extracts (Fig. 10).

### Effect of the hydromethanolic extract of *Ficus sycomorus* on the spontaneous alternation of rats in the Y-maze

The percentage of spontaneous alternation (spatial memory) of rats in the Y maze is represented by Fig. 11. In this test, one way ANOVA showed that there was a significant difference between the percentage of spontaneous alternation between the different group [F (4, 21) = 6.87; *P* = 0.00105]. The UCMS induced a decrease in spontaneous alternation if rat but the used of Newman-Keuls test indicated that the difference was not significant when compared to control group. On the other hand, for the animals treated with *F. sycomorus* extracts at doses of 100 mg/kg and 200 mg/kg and with imipramine (20 mg/kg), we noticed a significant increase (*p* < 0.01) of this parameter as compared to non-treated animals. At the same time ANOVA test indicated a significant difference between number of lines crossed by the animal in different groups [F (4, 24) = 5012, *P* = 0.00445]. Newman-Keuls post hoc comparison indicated that the effect of UCMS on the reduction of locomotor activity was significantly different from that of control animals in the Y-maze (*p* < 0.05). The locomotor activity of animals undergoing stress but treated with extract and imipramine and that of animals undergoing stress without treatment was no significant when compared.

### Effect of *Ficus sycomorus* extract on total content of reduced GSH and MDA levels

Rats of UCMS groups treated with both doses (100 and 200 mg/kg) of the hydromethanolic extract of *F. sycomorus* extract exhibited attenuation in lipid peroxidation, indicated by a significant decrease of MDA level [F (2, 20) = 4.267, *P* = 0.0117] (Fig. 12) estimated in the brain homogenates compared to control group. Post hoc analyses also revealed non-significant statistical differences (*p* > 0.05) between UCMS groups treated with the extract (100 mg/kg and 200 mg/kg). Furthermore, a significant increase of total GSH content [(F (2, 20) = 3.40, *p* = 0.028)] estimated in brain homogenates of UCMS groups exposed to the hydromethanolic extract of *F. sycomorus* compared to control groups, was observed. Additionally, post hoc analyses revealed no significant differences between UCMS and extract-treated nor imipramine groups.

In the other hand, the Pearson correlation calculation revealed that there was a significant and positive correlation between swimming time in the Forced swimming test vs. GSH (*r* = 0.624, *P* = 0.0015) (Fig. 13a) and between sucrose preference in the anhedonia test vs. GSH (*r* = 0.54, *P* = 0.0048) (Fig. 13b). At the same time, there were a negative but non-significant correlation between swimming time vs. MAD (*r* = 0.30, *p* = 0.17) (Fig. 13c) and between sucrose preference vs. MAD (*r* = 0.43, *P* = 0.29) (Fig. 13d).

The histological examination of the brain section did not revealed any significant abnormality or modification of the brain structure.

## Discussion

The present study investigated the effects of hydromethanolic extract of *F. sycomorus* stem barks on depression, anxiety and memory impairment induced by unpredictable chronic mild stress (UCMS) and on the oxidative stress status of the brain, known for its implication in neuropsychiatric disorders. Our results provide additional evidence regarding the involvement of the unpredictable chronic mild stress (UCMS) in the genesis of depression, anxiety and memory impairment by in rats and the role of the oxidative stress is the biological process.

The UCMS model of depression consist of the presentation of a series of varied and unpredictable environmental stressors. In the present study, as stressor, we used food and water deprivation, wet cages, isolation, forced swimming at 20 °C and flashing light. Following such exposure, animals have been reported to exhibit a persistent reduction in responsiveness to pleasurable stimuli, measured by a decrease in their consumption of 1% sucrose solution [36]. Reductions in sucrose consumption produced by UCMS procedure have been shown to be reversed by chronic treatment with either tricyclic antidepressants or Selective Serotonin Reuptake Inhibitors (SSRIs) [28].

During our study, animals in a normal psychological state showed a preference for the sucrose solution. However, after 30 days of UCMS there was a significant reduction of sucrose intake (*p* < 0.001), suggesting the loss of pleasure to all animals exposed to the stressor, in comparison with normal animals. These results are in accordance with those obtained by Willner et al. [37], and can be explained by an inactivation of the sub cortical regions of the brain as well as the cortex zones involved in reward and motivation. The treatment with *F. sycomorus* extracts has corrected this loss of pleasure for treated animals. In fact, the pre-treatment of animals subject to UCMS with *F. sycomorus* hydromethanolic extract at a dose of 100 mg/kg has caused a significant increase of sucrose consumption.

Anhedonia being known as a clinical manifestation of depression [38], these results suggest that the hydromethanolic extract of *F. sycomorus* possesses anti-depressive properties in rats. To confirm this hypothesis, the forced swimming test was used. This test is one of the most commonly used animal models for estimating the anti-depression effects of different classes of drugs in rodents [39].

This test is based on the observation that animals, after initial escape-oriented movements, develop an immobile posture when placed in an inescapable stressful situation. When antidepressant treatments are given prior to the tests, the subjects will actively persist engaging in escape-directed behavior for longer periods of time than after vehicle treatment [40]. In the present study, a significant antidepressant-like effect of the extract was observed at the dose of 100 mg/kg (*p* < 0.05). However this effect was less than that of imipramine (20 mg/kg), a tricyclic antidepressant used as standard drug in this test (*p* < 0.01).

Considering the monoaminergic hypothesis of depression we can thus suggest that the results described above may be explained by the fact that the UCMS impairment of monoaminergic neurotransmission, mainly adrenaline and serotonin and the treatment with the hydromethanolic extract of *F. sycomorus* improve the brain level of these neurotransmitters. In fact, it is clearly established that these neurotransmitters are involved in the expression of an antidepressant-like effect in behavioral despair models of depression [41]. The rats submitted to UCMS exhibited a longer latency to feed while the chronic administration of the extract at all the doses used, as well as imipramine significantly reduce the latency in feeding behavior when compared to the UCMS group (*p* < 0.01). This result can be explained by the antidepressant activity of the extract, but it is also well know that compounds with anxiolytic properties can induce positive response in this test, reducing the latency of the animals. In order to be more confident about the nature of the results, the same doses of the extract were used in the elevated plus maze test.

The elevated plus maze test is one of the most used tests to study compounds with anxiolytic effects on rodents [42]. It is based on the capacity of these rodents to avoid opened arms of the maze which, also in elevation is very anxiogenic, and to prefer closed arms which ensure security. After 30 days of UCMS, rats of the saline group (UCMS without any treatment) showed a significant increase on the time spent in the closed arm (*p* < 0.001), in comparison to control animals. These results suggest that the UCMS has effectively contributed in the development of anxiety in animals. This result has also been reported by Frih et al. [26] and may be due to the inhibitory activity of the GABAergic system [43]. On the other hand, the treatment of anhedonic rats with *F. sycomorus* extracts significantly reduced the number of entries in the closed arms (100 and 200 mg/kg, *p* < 0.001) and the number of time spent in the closed arms (100 mg/kg, *p* < 0.05). Concomitantly, this treatment has increased the number of entries in the opened arms and the time spent in these arms. These results suggest that the hydromethanolic extract of *F. sycomorus* may possess anxiolytic properties. These anxiolytic properties may be due to the action of compounds contained in the extract to interfere with the receptor complex of GABA and/or of the antagonistic action of these compounds on the receptors, causing an increase of the GABA activity [43]. Therefore, the between-group differences noted in the NFST may be also attributed to the anxiolytic effect of the extract.

In the elevated plus maze test, the number of rearing, of grooming and of head dipping have been registered. According to Augustsson [44], the reduction of these different parameters indicate a reduction in anxiety behavior. Our results on these three parameters confirm the conclusions taken above on the anxiogenic activity of UCMS and the anxiolytic activity of the methanolic extract of *F. sycomorus* bark. In fact, we have noticed a significant increase in the number of rearing (*p* < 0.01) and head dipping (*p* < 0.05) in the UCMS-non treated group in comparison to normal group. The treatment with the plant extract has caused a significant decrease of the number of rearing and head dipping (200 mg/kg, *p* < 0.01) while the effect of imipramine was more significantly important in the inhibition of the head dipping behavior (*p* < 0.001).

In the present study, the results showed that, only rats that experience chronic stress would show impaired performance in the Y-maze test. However those that were exposed to the UCMS and hydromethanolic extract of *F. sycomorus* at doses of 100 mg/kg and 200 mg/kg have significantly improved performance in comparison to stress alone (p < 0.01) in the same test. The effect of imipramine (20 mg/kg) in the prevention of this trouble was more significant than that of the plant extract (*p* < 0.001). These results clearly indicated that UCMS impair the spatial memory of rat and the hydromethanolic extract of *F. sycomorus* was able to prevent this deficit. However, at this level of our study, it is difficult for us to hypothesize that only rats exposed to UCMS could have their performance improved in Y-Maze or not. The specific effect of this extract on the memory remains to be determined by a more appropriate methodology.

This experimental model of the memory impairment was also experienced by Henningsen et al. [45]. In fact, a very close comorbidity exists between stress and neurologic damages. Stress causes at the endocrine level a modification of the circulating level of glucocorticoids such as cortisol, but equally an atrophy of the dendrites of the hippocampus and of the cortex, as well as high expression of the CRH [46]. This process at the end causes a disruption of the working memory as well as the reduction of the neurotransmitters activity involved in the process of memory [47]. Some receptors as NMDA receptors are involved in several processes in the central nervous system including synaptic plasticity, which reflects learning and memory. Antidepressant drugs could reverse the negative physiological effects of chronic stress and depression by either normalizing or increasing NMDA receptors, which play a role in learning and memory [48, 49].

It’s known that flavonoids are powerful antioxidants which act via the suppression of ROS (Reactive Oxygen Species) formation, either by enzyme inhibition or by chelation of oligo-elements involved in the generation of free radicals; by trapping of ROS and/ or rising the regulation of anti-oxidants [50]. This property of flavonoids may explain the beneficial effect of our extract on chronic stress as shown in the present study. Moreover, the LC-MS analysis, in positive mode (ES+) has shown that the major compound in the *F. sycomorus* hydromethanolic extract was a glycoside flavonoid, with the characteristics of the racemosic acid (C_22_H_28_O_14_). Racemosic acid has been isolated for the first time in *F. racemosa* [51]. To date, only its anti-oxidant and its anti-inflammatory properties by the inhibition of the Cox-1 and of 5-LOX is well established [51]. To the best of our knowledge, the results of this work indicate for the first time the protective effects of the methanolic extract of *F. sycomorus* on anxiety and mood disorders.

Our previous studies strongly suggest the central role of the oxidative stress in the neuropsychiatric disorders [8–10]. In the present study there was a decreased level of GSH and increased level of MDA in the rats that experienced chronic stress compared to normal rats. After 30 days, those that were exposed to the UCMS and treated with the hydromethanolic extract of *F. sycomorus* at doses of 100 mg/kg and 200 mg/kg showed an increase in the GSH level with a concomitant decrease of MDA in the brain homogenate. The involvement of ROS in the pathogenesis of depressive disorders was confirmed during this study by the positive and significant correlation established between the swimming time vs. GSH and between sucrose preferences vs. GSH, but also the negative correlation although not significant between Swimming time vs. MDA and Sucrose preference vs. MDA. These results suggest that the plant extract has an in vivo antioxidant activity and is capable of ameliorating the effect of ROS in the brain of rats. Grases et al. [52] also state that depression and anxiety are related to lowered plasma concentrations of antioxidants. Despite these results, some limitations need to be brought to the present work. Due to some technical difficulties we did not separate parts of brains and the biochemical estimations were done using the entire brain rather than the hippocampus. The expression of some protein markers involved in mental disorders was not done. These issues will be seriously taken into account in our upcoming investigations for better liability of the results.

## Conclusion

This study demonstrated the antidepressant-like effect of hydromethanolic extract of *F. sycomorus* in unpredictable chronic mild stress model of depression in male rats through sucrose preference test, novelty-suppressed feeding test and forced swimming test. The anxiolytic and spatial memory enhancing activity was also effective in EPM and Y-maze test respectively. Furthermore, the mechanism of the observed antidepressant-like effect was found to be mediated through the increase in total GSH and decrease in the lipid peroxidation (MDA level) in the rat temporal lobe homogenates. Racemosic acid, the major compound found in this extract, could be responsible for its pharmacological actions. Further investigations are absolutely necessary to evaluate the effect of this compound in various animal models of depression before any definitive conclusion.
