# Correction to: *Ficus sycomorus* extract reversed behavioral impairment and brain oxidative stress induced by unpredictable chronic mild stress in rats

**DOI:** 10.1186/s12906-017-2069-5

**Published:** 2017-12-28

**Authors:** Harquin Simplice Foyet, Serge Tchinda Deffo, Pascaline Koagne Yewo, Iulia Antioch, Stéphane Zingue, Emmanuel Acha Asongalem, Pierre Kamtchouing, Alin Ciobica

**Affiliations:** 1 Department of Biological Sciences, Faculty of Science, University of Maroua, Cameroon, P.O. Box: 814, Maroua, Cameroon; 2 Department of Life and Earth Sciences, Higher Teachers’ Training College, University of Maroua, Cameron, P.O. Box: 55, Maroua, Cameroon; 3 Department of Research, Faculty of Biology, Alexandru Ioan Cuza University, 11 Carol I Blvd, 700506 Iasi, Romania; 4 Department of Biomedical Sciences, Faculty of Health Sciences, University of Buea, Cameroon, P.O. Box 63, Buea, Cameroon; 5 Department of Animal Biology and physiology, Faculty of Science, University of Yaounde I, Yaounde, Cameroon

## Correction

After the publication of this article [1] it came to our attention that Harquin Simplice Foyet was incorrectly included as Harquin Simplice Harquin Foyet. The corrected name is included in the author list. The original article was updated.
